# Morphologic and Metabolic Comparison of Treatment Responsiveness with 18Fludeoxyglucose-Positron Emission Tomography/Computed Tomography According to Lung Cancer Type

**DOI:** 10.4274/mirt.36349

**Published:** 2016-06-06

**Authors:** Mehmet Fatih Börksüz, Taner Erselcan, Zekiye Hasbek, Birsen Yücel, Bülent Turgut

**Affiliations:** 1 Aksaray State Hospital, Clinic of Nuclear Medicine, Aksaray, Turkey; 2 Muğla Sıtkı Koçman University Faculty of Medicine, Department of Nuclear Medicine, Muğla, Turkey; 3 Cumhuriyet University Faculty of Medicine, Department of Nuclear Medicine, Sivas, Turkey; 4 Cumhuriyet University Faculty of Medicine, Department of Radiation Oncology, Sivas, Turkey

**Keywords:** Standardized uptake value, total lesion glycolysis, metabolic tumor volume, Lung Cancer, positron emission tomography/computed tomography, treatment response

## Abstract

**Objective:**

The aim of the present study was to evaluate the response to treatment by histopathologic type in patients with lung cancer and under follow-up with 18F-fluoro-2deoxy-glucose-positron emission tomography/computed tomography (18F-FDG PET/CT) imaging by using Response Evaluation Criteria in Solid Tumors (RECIST) and European Organisation for Research and Treatment of Cancer (EORTC) criteria that evaluate morphologic and metabolic parameters.

**Methods:**

On two separate (pre- and post-treatment) 18F-FDG PET/CT images, the longest dimension of primary tumor as well as of secondary lesions were measured and sum of these two measurements was recorded as the total dimension in 40 patients. PET parameters such as standardized uptake value (SUVmax), metabolic volume and total lesion glycolysis (TLG) were also recorded for these target lesions on two separate 18F-FDG PET/CT images. The percent (%) change was calculated for all these parameters. Morphologic evaluation was based on RECIST 1.1 and the metabolic evaluation was based on EORTC.

**Results:**

When evaluated before and after treatment, in spite of the statistically significant change (p<0.05) in SUVmax, the change was not significant in TLG, in the longest total size and in the longest size (p>0.05). In histopathologic typing, when we compare the post-treatment phase change with the treatment responses of RECIST 1.1 and EORTC criteria; for RECIST 1.1 in squamous cell lung cancer group, progression was observed in sixteen patients (57%), stability in seven patients (25%), partial response in five patients (18%); and for EORTC progression was detected in four patients (14%), stability in thirteen patients (47%), partial response in eleven patients (39%), in 12 of these patients an increase in stage (43%), in 4 of them a decrease in stage (14%), and in 12 of them stability in stage (43%) were determined. But in adenocancer patients (n=7), for RECIST 1.1, progression was determined in four patients (57%), stability in two patients (29%), partial response in one patient (14%); for EORTC, progression in one patient (14%), stability in four patients (57%), partial response in two patients (29%) were observed and in these patients, an increase in stage was detected in 3 of them (43%), while 4 of them remained stable. According to histopathologic diagnosis, between squamous cell cancer and adenocancer cases, no significant difference was determined in terms of SUVmax (p>0.05). Post-treatment SUVmax was significantly different in primary tumor but was not significantly different in nodal involvement and metastatic lesions for squamous cell carcinoma patients as compared to the pre-treatment SUVmax measurements. Similarly, there was no significant difference between primary tumor and nodal involvement for adenocarcinoma patients.

**Conclusion:**

Whether metabolic or morphologic changes are more accurate in evaluating treatment response in lung cancer remains unknown, and there is no gold standard diagnostic method on this issue yet. The most reliable results can only be achieved by survival curve parameters. However, we believe SUVmax seems to provide more easy and practical data for the evaluation of treatment response.

## Abstract (tr)

**Amaç:**

Bu çalışmadaki amaç 18F-floro-2deoksi-glikoz-pozitron emisyon tomografisi/bilgisayarlı tomografi (18F-FDG PET/BT) ile takip edilen akciğer kanseri hastalarında histopatolojik hücre tipine göre tedavi yanıtını, morfolojik ve metabolik parametreleri dikkate alan Solid Tümörlerde Cevap Değerlendirme (RECİST) ve Avrupa Kanser Araştırma ve Tedavi Organizasyonu (EORTC)kriterleri ışığında değerlendirmektir.

**Yöntem:**

Kırk hastanın çekilen iki ayrı (tedavi öncesi ve sonrası) 18F-FDG PET/BT tetkikindeki primer tümörün ve eş zamanlı ikincil lezyonların en uzun boyutları ölçüldü ve bu ölçümler toplanarak ‘toplam boyut’ olarak kaydedildi. Hedef alınan bu lezyonların standart tutulum değeri (SUVmaks), metabolik tümör volümü ve toplam lezyon glikolizis (TLG) gibi PET parametreleri tedavi öncesi ve sonrası iki ayrı 18F-FDG PET/BT tetkikinde kaydedildi. Bu verilerin tedavi öncesine göre yüzde (%) değişimi her hasta için ayrı ayrı hesaplandı. Morfolojik değerlendirme RECİST 1.1, metabolik değerlendirme ise EORTC kriterlerine göre yapıldı.

**Bulgular:**

Tedavi öncesi ve sonrası değerlendirildiğinde, SUVmaks’taki istatistiksel olarak anlamlı (p<0,05) değişime karşın, en uzun boyut, en uzun toplam boyut ve TLG’deki değişim anlamlı değildi (p>0,05). Histopatolojik tiplendirmede, RECİST 1.1 ve EORTC kriterlerine göre tedavi yanıtlarını tedavi sonrası evre değişimi ile arşılaştırdığımızda; RECİST 1.1’e göre skuamoz hücreli akciğer kanseri grubunda on altı hastada progresyon (%57), yedi hastada stabilite (%25), beş hastada parsiyel cevap (%18), EORTC’a göre dört hastada progresyon (%14), on üç hastada stabilite (%47), on bir hastada parsiyel cevap (%39) izlenirken, bu hastaların on ikisinde evrede artış (%43), dördünde evrede azalma (%14) ve on ikisinde evrede stabilite (%43) saptandı. Adenokanserli hasta grubunda (n=7) ise RECİST 1.1’e göre dört hastada progresyon (%57), iki hastada stabilite (%29), bir hastada parsiyel cevap (%14); EORTC’a göre bir hastada progresyon (%14), dört hastada stabilite (%57), iki hastada parsiyel cevap (%29) izlenirken, bu hastaların üçünde evrede artış (%43), dördünde ise evrede stabilite saptandı. Histopatolojik tanıya göre skuamöz hücreli kanser ve adenokanser olguları arasında SUVmaks değerlerinde anlamlı farklılık saptanmadı (p>0,05). Skuamöz hücreli kanserde primer tümörde tedavi sonrası SUVmaks değişimi anlamlı iken, nodal tutulumda ve metastatik lezyondaki değişimde ise anlamlı farklılık saptanmadı. Benzer şekilde, adenokanser hastalarında da primer tümörde ve nodal tutulumda anlamlı farklılık saptanmadı.

**Sonuç:**

Akciğer kanserinde tedavi yanıt değerlendirmede metabolik ve morfolojik değişikliklerden hangisinin daha doğru sonuç verdiği kesin olarak bilinmiyor olup bu konuda altın standart bir tanı yöntemi de henüz yoktur. En doğru sonuçlar ancak yaşam eğrisi parametreleri ile gösterilebilir. Ancak, SUV ölçümünün tedavi yanıtını takipte daha kolay ve pratik bilgi verdiğini düşünüyoruz.

## INTRODUCTION

Lung cancer is the most common cancer in men and the fifth cancer in women, with 53300 new male cases per year (1). The majority of lung cancer is non-small cell lung cancer (NSCLC) tumors which consist of subtypes such as adenocarcinoma, squamous cell carcinoma, large cell carcinoma and carcinoid tumor (2). 18F-fluoro-2-deoxy-glucose positron emission tomography/computed tomography (18F-FDG PET/CT) is widely used throughout the world in lung cancer for primary diagnosis, staging, restaging, evaluation of treatment response and radiotherapy (RT) planning (3). The maximum standardized uptake value (SUVmax) is widely recognized as an adequate imaging biomarker for the prognosis of lung cancer (4). A SUV of >2.5 is considered as evidence of malignancy in solitary lung nodules. However, lesions smaller than twice the resolution of imaging systems usually yield underestimated SUV values (5). Moreover, SUV may be lower than 2.5 in bronchoalveolar carcinoma involving no other histological component (6). Parameters such as SUVmax have been used for diagnosis and evaluation of treatment effectiveness in lung cancer. In addition, metabolic parameters such as metabolic tumor volume (MTV) and total lesion glycolysis (TLG) can also be estimated by 18F-FDG PET/CT that have been considered as prognostic factors in patients with NSCLC, independent of tumor-node-metastasis stage (7). MTV represents the three-dimensional total volume within the region of interest drawn around the lesion. The highest SUV (SUVmax) and the average SUV (SUVmean) measured within this volume can be estimated. TLG value for the lesion, which is directly related to these two measurements, is calculated as follows: “TLG=MTVxSUVmean” (8). The Response Evaluation Criteria in Solid Tumors (RECIST) criteria are being used for the morphologic evaluation of the response to treatment in lung cancer, while the metabolic response is being evaluated by the European Organization for Research and Treatment of Cancer (EORTC) criteria. Since the introduction of 18F-FDG PET/CT in routine clinical practice, studies on the Positron Emission Tomography Response Criteria in Solid Tumors (PERCIST), which is the criteria of tumor response as related to 18F-FDG-PET, are being conducted. PERCIST suggests using lean body mass-normalized value instead of SUV (the activity concentration in tumor/injected dose/patient weight). The aim of the present study was to assess treatment response according to histological types in lung cancer patients by using RECIST and EORTC criteria, which evaluate morphologic and metabolic parameters.

## MATERIALS AND METHODS

A total of forty patients (38 males, two females, median age=63.3±6 years; range=46-73) who underwent PET/CT were included in the study. In the initial assessment, there was a mixed population in whom primary staging had been done and the treatment had been given. 18F-FDG PET/CT was performed to assess treatment response following chemotherapy or chemoradiotherapy. PET imaging was performed using a combined PET/CT scanner (Discovery 600 PET/CT GE Medical Systems, USA). Each patient fasted for at least 6 h before imaging. After ensuring that blood glucose was <150 mg/dl, approximately 370 MBq 18F-FDG were administered i.v. 1 h before image acquisition. Attenuation correction of PET images with the CT data was performed. The CT scan was performed first. Right after CT data acquisition, a standard PET imaging protocol was taken from the cranium to the mid-thigh with an acquisition time of 3 min/bed in 3-dimensional mode. CT and PET images were matched and fused into transaxial, coronal and sagittal images. The data were transferred via the Digital Imaging and Communications in Medicine protocol to a processing Workstation (AW Volumeshare 5 GE Medical Systems S.C.S, France). The visual and semi-quantitative analyses were then performed. The longest dimension of the primary tumor on two separate 18F-FDG PET/CT images were measured in the mediastinum window on CT. Moreover, “total size” was calculated by summing the longest dimensions of the two lesions with maximum size or of any five lesions in an organ (lung). For the lymph nodes, the short axis measurement was also included in this measurement. Two separate 18F-FDG PET/CT images obtained in the pre-treatment and post-treatment periods were assessed and the SUVmax, SUVmean and MTV of the target lesions were recorded. The percent change in the longest size of the primary tumor and total size as well as in SUVmax, SUVmean and TLG was calculated for each patient in comparison to the pre-treatment values. Morphologic assessment was made according to RECIST 1.1 criteria by considering the percent change in the total longest dimension of the target lesions in the post-treatment period. Metabolic assessment was made according to EORTC criteria by calculating the percent change in SUVmax of the primary tumor in the post-treatment period.

### Statistical Analysis

Data were analyzed by using SPSS version 14.0 and expressed as mean±standard deviation. The pre-treatment and post-treatment dimensions measured on CT, and SUVmax and TLG values on PET were compared by the paired T-test. Among the histopathologic diagnosis of the patients, the two major groups of patients with a diagnosis of squamous cell carcinoma (n=28/40) or adenocarcinoma (n=7/40) were compared in terms of SUVmax values by using Kruskall Wallis analysis. Moreover, the percent change in post-treatment longest dimension, SUVmax and TLG (increased or decreased) were compared by using chi-square (Fisher) test. Significance level was set at p<0.05.

## RESULTS

Of the forty patients included in the study, 2 were female (5.0%) and 38 were male (95.0%) with a mean age of 63.3±6 years (range, 46-73 years). In terms of histopathologic diagnosis, 28 patients had squamous cell lung carcinoma (70%), seven had adenocarcinoma (17.5%), four had small cell cancer (10%) and one had pleomorphic cell lung cancer (2.5%) (Table 1). Mean follow-up time was 23.1±12.6 weeks (range, 10-67 weeks). The treatment methods were separate RT+chemotherapy (CT) sessions in two patients, chemoradiotherapy in three patients and only CT in the remaining 35 patients. Nine patients had distant metastatic lesions in addition to the primary lesion and lymph node involvement, and the metastasis was measurable in seven of these patients. According to the histopathologic diagnosis, pre-treatment SUVmax was 16.1±6.9 (n=28) vs. 20.4±14.1 (n=7) in patients with squamous cell carcinoma and adenocarcinoma, respectively (p>0.05). Post-treatment change in SUVmax was found to be statistically significant in patients with squamous cell carcinoma. However, there was no statistical difference in the FDG uptake change for patients with lymph node involvement or metastatic lesions. Similarly, there was no significant difference in primary tumor and nodal involvement in the comparison of adenocarcinoma patients. Pre- and post-treatment longest dimension, SUVmax and TLG of the primary tumor were compared in twenty-eight patients with a histopathologic diagnosis of squamous cell carcinoma who constitute the majority of the patients in order to assess treatment response. Mean SUVmax was significantly different between pre- and post-treatment measurements (p<0.05), with no difference in terms of longest dimension, total dimension and mean TLG (p>0.05). The pre- and post-treatment longest dimension, SUVmax and TLG were also compared in adenocarcinoma patients. Patients with adenocarcinoma (n=7) had no significant difference in these parameters measured before and after the treatment (p>0.05) (Table 2). Table 3 represents the pre-treatment and post-treatment SUVmax change in the primary tumor, lymph node and metastatic lesions stratified by histopathologic diagnosis. When the response to treatment was compared in patients with squamous cell cancer according to RECIST 1.1 and EORTC criteria by post-treatment stage change, RECIST 1.1 revealed progression in sixteen patients (57%), stability in seven (25%) and partial response in five (18%); while EORTC revealed progression in four (14%), stability in thirteen (47%) and partial response in eleven (39%) patients (Table 4). Of these patients, twelve (43%) showed increased stage, 4 (14%) had decreased stage with the remaining 12 (43%) at a stable stage. In patients with adenocarcinoma (n=7), RECIST 1.1 revealed progression in four (57%), stability in two (29%), and partial response in one (14%) patient; while EORTC showed progression in one (14%), stability in four (57%), and partial response in two (29%) patients. Of these patients, 3 (43%) showed increased stage and the remaining 4 had a stable stage. Data on change in size and metabolic parameters and rates (%) according to histopathologic type of primary tumor are presented in Table 2. In our study, PET/CT and CT data of four patients diagnosed with small cell lung cancer before and after treatment were compared and the following conclusion was reached: Although the average SUVmax decreased, two patients showed metastatic progression and upstaging. And one patient with the diagnosis of stage 4 pleomorphic Ca according to pre-treatment PET/CT, showed an increase in size and TLG at post-treatment PET/CT.

## DISCUSSION

18F-FDG PET/CT is used for the diagnosis and staging of lung cancer as well as for the assessment of response to treatment. In the present study, we investigated the association between the morphologic features (dimension) and metabolic criteria for assessment of response to treatment (SUV and TLG). One of the first studies by Kubota et al. (9) assessing the metabolic and morphologic comparison of response to treatment in patients with lung cancer was carried out with radiopharmaceutical 11C L-methionine, which is a marker for protein synthesis and cell proliferation. Change in dimension has been assessed by CT and the change in nodal uptake has been measured by PET not using a hybrid device. The outcome has been divided into 3 groups of early progression, late local recurrence and no local recurrence. Methionine uptake was decreased by 72% and 65% in the groups of late local recurrence and no local recurrence at PET imaging obtained 2 weeks after RT, while it was found to decrease by 22% in the group of early progression. The authors have concluded that PET imaging was more beneficial in predicting local recurrence and progression as compared to CT imaging. Kubota et al. (9) have carried out PET and CT imaging methods on separate devices. In contrast to these studies, we used an integrated PET/CT device. Although there was no significant change in the longest dimension of the primary tumor and in total target dimension and TLG of the primary tumor, the SUVmax showed a statistically significant change after the treatment in patients with squamous cell carcinoma as well as in the whole group. Another study by Patz et al. (10) assessing the response to treatment only with PET imaging included 113 patients treated with chemotherapy, RT, surgery or a combination of these modalities. The authors have evaluated the examinations performed within an average of 8 months after the treatment, and have found that the PET imaging was negative in 13 vs.100 positive patients. In our study, two of the forty patients died in the last evaluation time. For this reason, in the present study, we did not perform a survival analysis. Another study has evaluated the patients treated with only chemotherapy and has aimed to predict the final outcome on PET imaging obtained after the first course of chemotherapy. In that study carried out on seven patients by Weber et al. (11), median survival time was 151 days in patients with more than 20% decrease in FDG SUV vs. 54 days in those without. The authors have also suggested a close relationship between assessment criteria for response to treatment and metabolic response in solid tumors. Cerfolio et al. (12) have evaluated 56 patients with NSCLC by 18F-FDG-PET within 1 month after neoadjuvant chemotherapy or combined RT before surgery. The authors have found a correlation between the change in SUVmax and percent rate of non-living cell number (%) during the resection. It was reported that the overall pathological response could be predicted with 96% accuracy when an 80% decrease in SUVmax was considered as the threshold value.

Pöttgen et al. (13), on the other hand, have performed resection to patients in whom PET images were obtained about 63 days after 3 courses of induction chemotherapy and 84 days after combined RT. There was an average of 67% decrease in SUVmax in patients treated with induction chemotherapy, with no or less than 10% living cancer cells in the resection. Moreover, patients having more than 10% living cancer cells had a mean decrease of 34% in SUVmax. In the present study, in the post-treatment evaluations, 22 patients had an average decrease of 25.4±15.8 mm in the longest dimension of the primary tumor while the remaining eighteen patients had an average increase of 30.6±28.6 mm in the longest dimension of the primary tumor. When the target lesions were also included in these measurements according to the RECIST 1.1 criteria, 22 patients had an average decrease of 24.8±16.1 mm and eighteen patients had an average increase of 39.2±44 mm. According to the RECIST 1.1 criteria, when the new lesion formation and the increase in unmeasurable lesions were also included in these measurements, eight patients had partial response, 22 had progressive disease and 10 had a stable disease. In the evaluations by using the SUVmax values based on EORTC criteria, SUVmax decreased by 38.9±25.6% in twenty-seven patients. In the remaining thirteen patients, there was an increase by 23±17.8%. Overall, sixteen patients had partial response, 19 had a stable disease and 5 had a progressive disease. Use of the changes in post-treatment longest dimension and SUVmax for the evaluation of response to treatment yields different results. In the present study, patients were evaluated according to the metabolic and morphologic features separately and the response to treatment differed with the use of RECIST 1.1 and EORTC criteria. With today’s technology, there is no gold standard diagnostic method to be used to determine which one is more accurate in evaluating the response to treatment. It is also not possible to evaluate all suspicious lesions with histopathologic methods. Therefore, response to treatment should be evaluated by comparisons with survival curves. However, FDG uptake is associated with living cancer cells and SUV increases with the increasing FDG uptake. Therefore, FDG uptake may be useful in the differentiation of tumor tissue, fibrosis and necrosis for which anatomical boundaries are not always distinguishable. Ordu et al. (14) reported in their study that, in advanced NSCLC patients, in evaluation of response to chemotheraphy and in determination of overall survival, the metabolic response with PERCIST may be an early predictive factor in comparison with morphologic response. Our study showed that although there was no significant change in the longest total dimension, the change in post-treatment SUVmax was significant whether only the longest dimension of the primary tumor or of target lesions were taken into account. There were seven patients who met the criteria of longest total dimension for the evaluation of metastatic disease (n=7/9). The longest total dimension in metastatic disease did not differ significantly between pre- and post-treatment periods. However, average SUVmax in metastatic lesions and nodal involvement differed significantly between pre- and post-treatment periods. As in the primary tumor, the response of metastatic lesions was also different between pre- and post-treatment periods in terms of dimension (morphologic feature) and SUVmax (metabolic feature). Overall, dimension criteria tended to be negative for the patient (unresponsiveness to treatment). Treatment response criteria should be standardized in a way that can be easily used in clinical practice for the patient examination reports. However, response criteria usually are not specified in the imaging methods for cancer patients (both in CT and PET/CT). This may result in difficulty for daily clinical practice. Moreover, although lymph nodes are included in RECIST 1.1, it is not always possible to determine the post-treatment changes in lymph nodes that are close to each other (and in conglomerated nodes). After the treatment period, a lymph node may have a decrease in size while the size of the next lymph node might have been increased. Because these nodes do not have clear borders, it is quite difficult to evaluate the treatment response in lymph nodes. In addition, RECIST 1.1 basically considers the tumor and the lymph node size. However, recent targeted anticancer drugs inhibit the growth of cells without killing the tumor cells. Thus, responders may display morphologic changes such as necrosis, cavitation, and hemorrhage in tumor without change in size (15). In this context, a more practical and easily used method, the metabolic criteria can be preferred.

## CONCLUSION

In this study on lung cancer patients, there was no significant difference between squamous cell and adenocancer group in terms of primary tumor SUVmax rates. There was no significant difference between pre- and post-treatment measurements in the longest dimension of primary tumor and in the total longest dimension selected as a target lesion. There was a significant SUVmax change after the treatment as compared to that of prior to the treatment. In the determination of treatment response, it is not known exactly yet whether metabolic or morphologic changes as evaluated by RECIST 1.1 and EORTC is more accurate in determining treatment response is yet unknown. Unfortunately, we could not comment on this issue because we had a limited number of patients and follow-up period. At the same time, we think that SUV rates can be preferred for treatment response evaluation due to its easier applicability in clinical practice.

## Ethics

Ethics Committee Approval: The study were approved by the Cumhuriyet University of Local Ethics Committee, Informed Consent: Consent form was filled out by all participants.

Peer-review: Externally peer-reviewed.

Financial Disclosure: The authors declared that this study has received no financial support.

## Figures and Tables

**Table 1:**
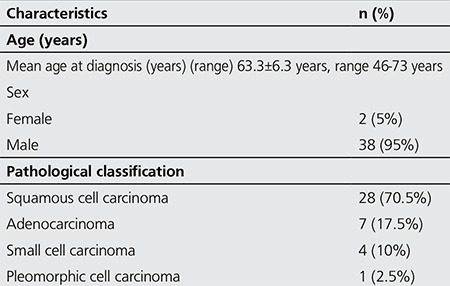
Demographic and clinico-histopathologic characteristics

**Table 2:**
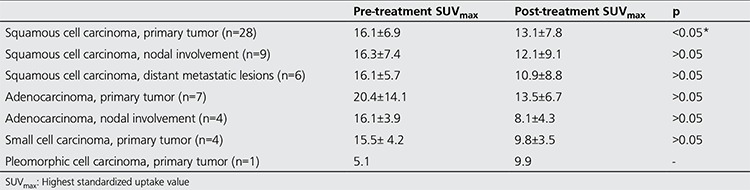
Pre-treatment and post-treatment highest standardized uptake value change in the primary tumor, lymph node and metastatic lesions according to histopathologic diagnosis

**Table 3:**
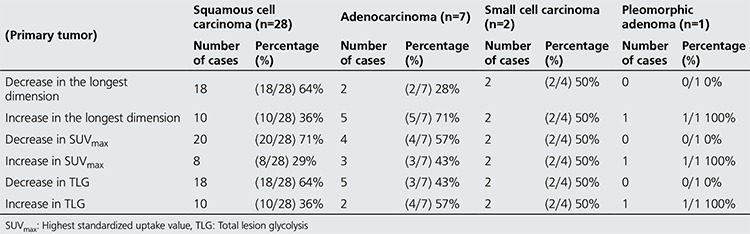
The number of cases with change in size and metabolic parameters according to histological type and the percent change (%)

**Table 4:**
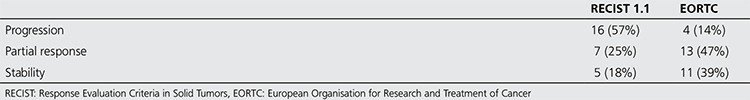
The treatment responses of squamous cell cancer patients according to Response Evaluation Criteria in Solid Tumors 1.1 and European Organisation for Research and Treatment of Cancer criteria
